# A giant neobladder stone with insignificant symptoms: A case report and literature review

**DOI:** 10.3389/fsurg.2023.1105146

**Published:** 2023-02-16

**Authors:** Jun Gu, Zexi He, Haihao Li, Yijie Liu, Haifeng Wang, Yinglong Huang, Mingxia Ding

**Affiliations:** Department of Urology, The Second Affiliated Hospital of Kunming Medical University, Kunming, China

**Keywords:** radical cystectomy, neobladder, neobladder stones, cystolithotomy, case report, orthotopic ileal neobladder

## Abstract

**Background:**

Giant neobladder lithiasis after orthotopic bladder replacement is an infrequent but important long-term complication, which should be diagnosed and treated early. If left untreated, it may eventually lead to irreversible acute kidney injury and seriously affect the quality of life of patients. Here, we present a rare case of a patient who presented with a massive neobladder stone after radical cystectomy done with orthotopic neobladder construction, followed by a challenging stone extraction process.

**Case presentation:**

A 70-year-old female patient presented with a massive neobladder stone 14 years after radical cystectomy done with orthotopic neobladder construction. A computed tomography scan showed a large elliptic stone. The patient underwent suprapubic cystolithotomy surgery, which removed her giant-sized stone in the neobladder. The size of the bladder stone that was removed was 13 cm × 11.5 cm × 9 cm, with a total weight of 903 g. To date, the follow-up time of treatment is 4 months, and in our patient, no pain, urinary tract infections, or other abnormalities suggestive of fistula were found.

**Conclusion:**

Imaging examination is useful for detecting neobladder lithiasis occurring after orthotopic neobladder construction. Our experience demonstrates that open cystolithotomy is an appropriate therapeutic method for treating the late-stage complication of a giant neobladder stone.

## Introduction

The standard treatment for muscle-invasive bladder cancer and high-risk non-muscle-invasive bladder cancer is radical cystectomy and urinary diversion (1–4). Compared with other urinary diversion methods, orthotopic neobladder construction is more effective, and can maximally restore the voluntary urination ability of patients and significantly improve their quality of life (5). However, it inevitably leads to some early and late complications. Neobladder lithiasis following this procedure, is a well-known long-term complication, but the factors leading to stone formation are complex and incompletely understood. Herein, we report a rare case of a patient with a giant neobladder lithiasis (>10 cm) located in the orthotopic ileal neobladder that was successfully removed by performing suprapubic cystolithotomy surgery.

## Case presentation

**Figure 1:**
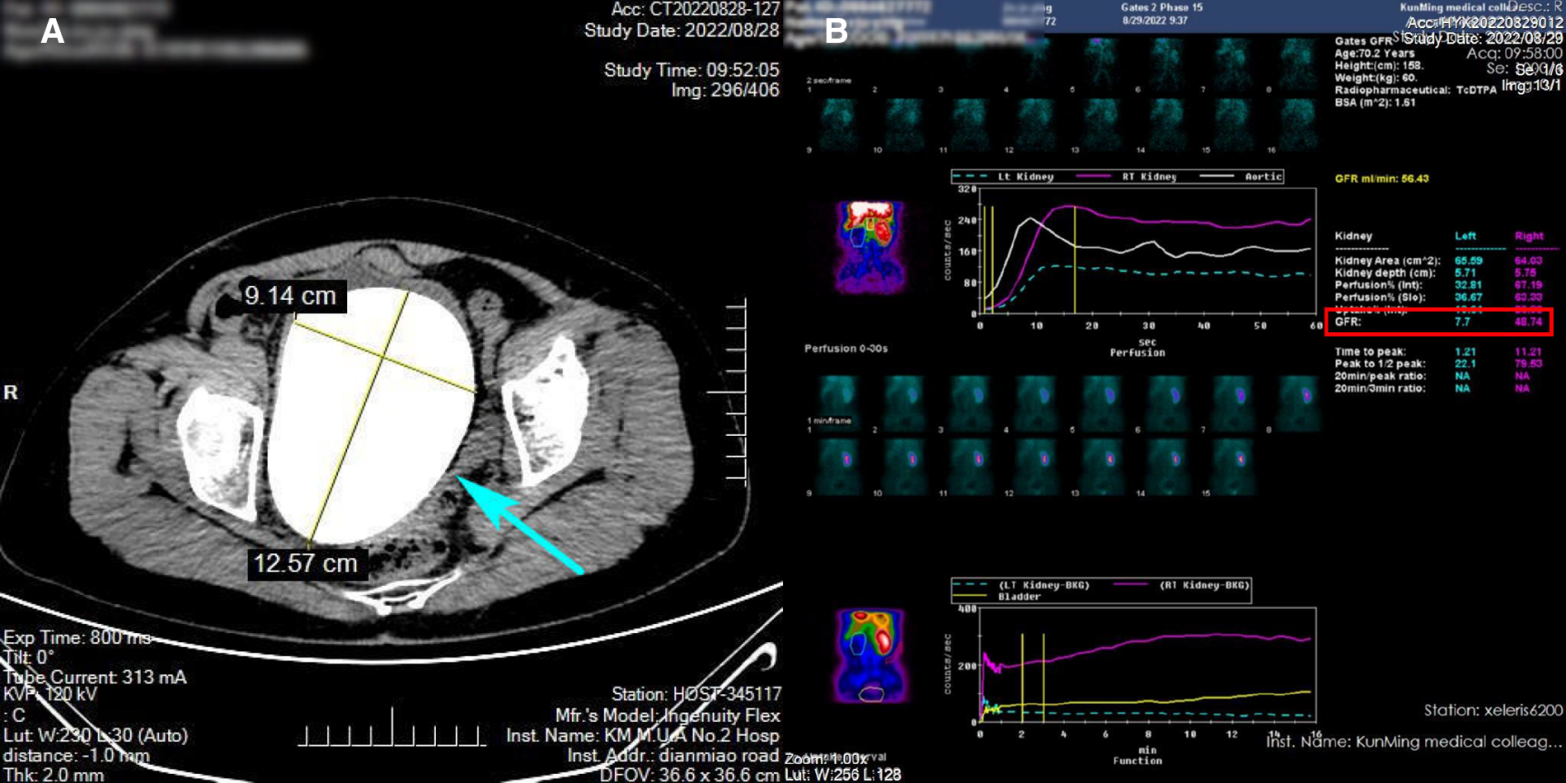
Computed tomography scan showing a neobladder stone (**A**, blue arrow). A renal scan showing poor blood perfusion in the left kidney (**B**).

**Figure 2:**
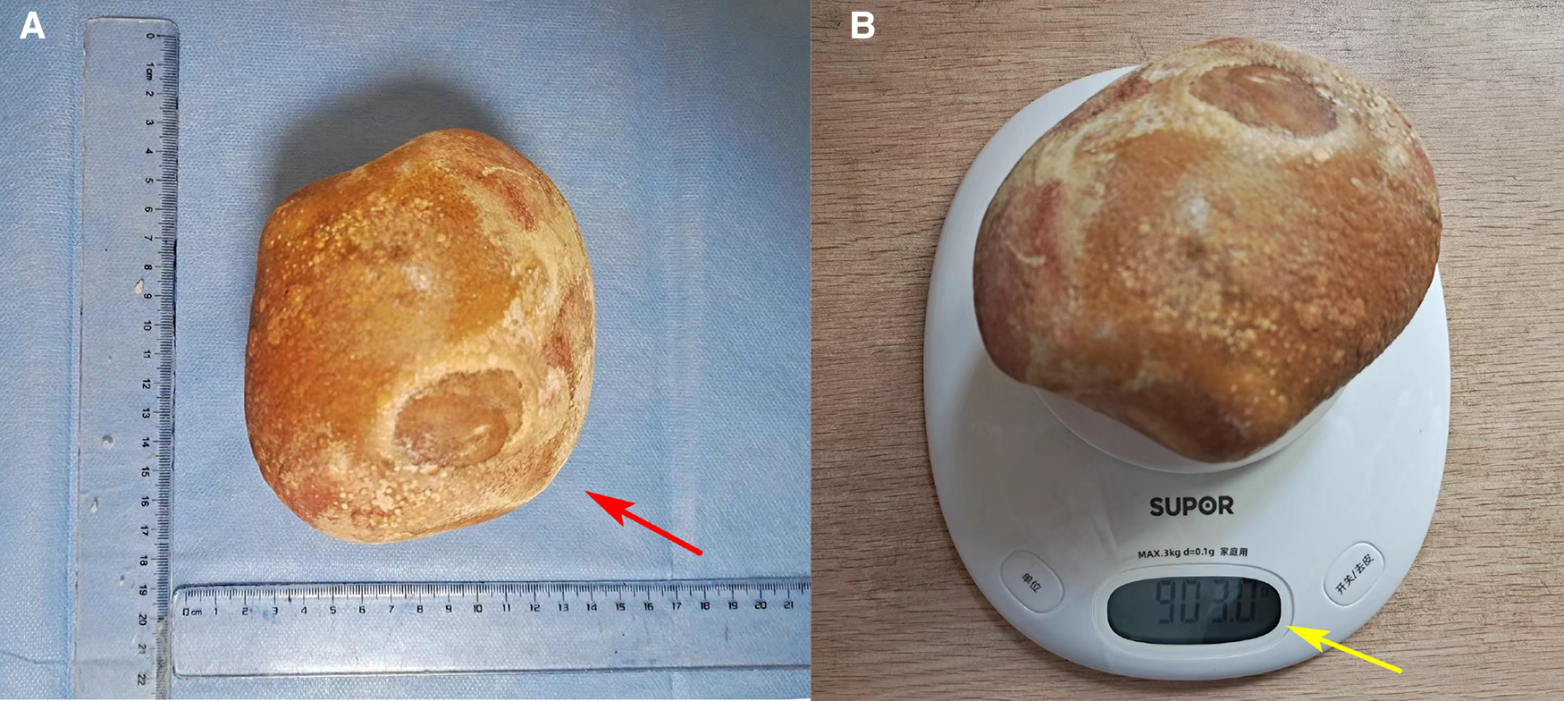
The size (**A**, red arrow) and weight (**B**, yellow arrow) of neobladder stones.

**Figure 3:**
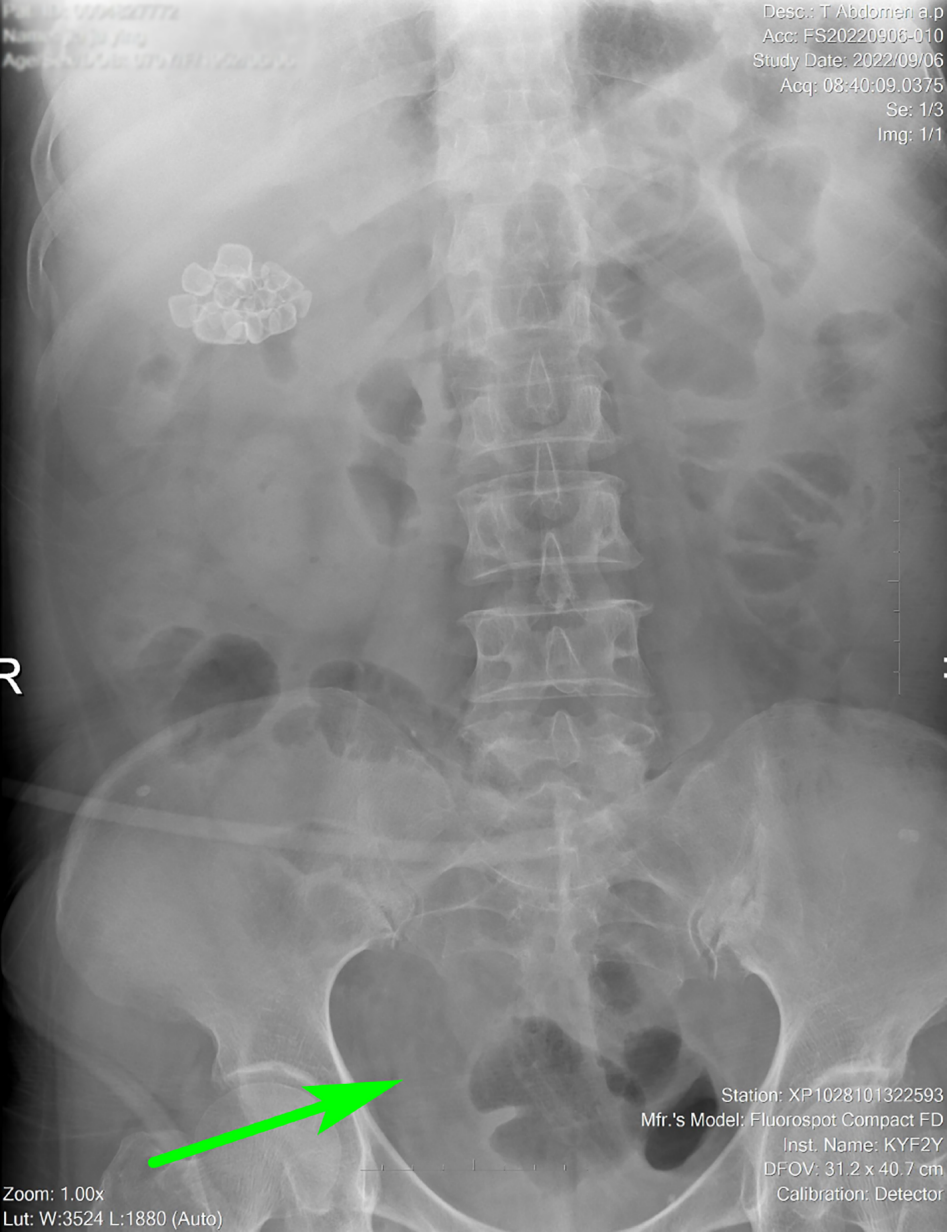
Postoperative abdominal x-ray showing no stones (green arrow).

A 70-year-old female patient was admitted to the hospital with a 1-week history of hematuria and a 3-day history of oliguria. Fourteen years ago, she underwent radical cystoprostatectomy and orthotopic ileal neobladder for pT2N0M0 bladder transitional cell carcinoma. The patient complained of dysuria to a certain extent and urine leakage after operation, requiring one to two perineal pads during the day and one at night. However, she did not continue follow-up regularly for 4 years after surgery because she did not experience any serious discomfort. A B-ultrasound examination revealed a thickened neobladder wall and a strong echo mass in the new bladder with an acoustic shadow (estimated initially to be approximately 93 mm × 105 mm in size), suggesting the possibility of calculi. A computed tomography (CT) revealed severe hydronephrosis in the left kidney and a huge high-density stone formation at the level of the neobladder (approximately 125 mm × 105 mm × 90 mm in size) that rendered the process of emptying of the neobladder difficult (Figure 1A, blue arrow). After admission, a physical examination showed an old surgical scar in the lower abdomen and a hard, unfixed uplift in the bladder zone, approximately 150 mm × 140 mm in size, without tenderness, rebound tenderness, or any other positive signs. A renal function test showed that creatinine was 105 μmol/L and the estimated glomerular filtration rate (eGFR) was 46 mL/min. A renal scan demonstrated poor blood perfusion in the left kidney (GFR 7.7 mL/min) and normal blood perfusion in the right kidney (GFR 48.74 mL/min) (Figure 1B). Urine analysis and urine cytology showed leukocyte esterase (3+) and urinary leukocyte (775.3 μL), suggesting evidence of urinary tract infection. The result of a urine culture test suggested the presence of *Escherichia coli* in the urine. Therefore, we controlled the urinary tract infection by employing a drug susceptibility test. After placing a Foley catheter to guide the position of the neobladder, suprapubic cystolithotomy was performed. An intraoperative exploration revealed dense intra-abdominal adhesions and a huge filled calculus in the neobladder. After cautious separation along the neobladder wall to avoid dissociating too much bowel, we found that the neobladder stones were huge and hard, and there was a lot of mucus, which resulted in limited surgical space and a lack of good force points. Therefore, we faced great difficulties in the process of removing the giant neobladder stone. Fortunately, we found that the size and shape of the new bladder stones were similar to those of the skull of a newborn. The two lobes of the forceps were able to hold the new bladder stone in a very small space. Ultimately, we creatively used the forceps during the operation and successfully removed the stone. The size of the stone was approximately 13 cm × 11.5 cm × 9 cm (Figure 2A, red arrow) and the weight was approximately 903 g (Figure 2B, yellow arrow). The color of the stone was brownish yellow and it was hard. Careful irrigation was performed and the pelvic cavity was then placed on a drainage tube. A postoperative abdominal x-ray showed absolutely no residual stones (Figure 3, green arrow). A urinary fistula developed at the neobladder cystotomy repair site, which was successfully resolved after the placement of pelvic and Foley catheters. After 4 months of follow-up, the patient became free of any pain, urinary tract infections, or other abnormalities suggestive of fistula.

## Discussion and conclusion

Radical cystectomy with urinary diversion remains an effective method for treating muscle-invasive bladder cancer. Orthotopic neobladder construction is a more effective urinary diversion method than others, with a high postoperative quality of life; it has been widely used in clinical practice (2–4). The orthotopic neobladder reconstruction technique alters the normal postoperative anatomy. Therefore, the group of patients who undergo this special procedure often have some complications in the early or late postoperative period, such as urine leakage, bowel obstruction, effusion, neobladder rupture, hydronephrosis, urinary tract infection, vesicoureteral reflux, urinary calculi, and tumor recurrence. (6). Among these complications, urolithiasis is a recognized late-stage complication of orthotopic neobladder reconstruction.

Through a comprehensive search of the PubMed database, we found that the overall incidence of urolithiasis varies greatly by different studies and the type of diversion. Miyake et al. reported 5 cases of neobladder stones among 80 consecutive Japanese patients with radical cystectomy and orthotopic sigmoid neobladder (5/80, 6.25%) (7). Moeen et al. observed that nine patients suffered from neobladder calculi among 197 patients who underwent radical cystectomy and orthotopic ileal neobladder reconstruction between the years 2007 and 2013 (9/197, 4.56%) (8). Hautmann et al. analyzed the long-term complications in a large, single-center series of patients who underwent cystectomy and substitution with an ileal neobladder and found that only 2 of the 923 patients developed reservoir stones (9). Also, the incidence of urolithiasis ranged from 16.7% to 43.1% in the case of ileal neobladder with a Kock pouch (10, 11).

In general, we find that neobladder stones after urinary diversion may be asymptomatic and are often discovered incidentally upon a radiological examination. Due to this, neobladder stones can grow to staggering sizes in the absence of a long-term follow-up, as shown in our patient case. However, to the best of our knowledge, few stones larger than 10 cm have been recorded in patients with a urinary diversion. However, a subset of patients may present with severe clinical symptoms such as lower abdominal pain, frequent urination, dysuria, and hematuria. Interestingly, Abrol et al. (12) reported an unusual clinical presentation of left medial thigh pain in a patient with a sigmoid neobladder, which may be attributed to obturator neuropathy caused by a compression of the nerve or nerve root between the psoas muscle and the stone.

At present, the chemical composition of neobladder stones reported in the literature is essentially the same; they are mainly composed of magnesium ammonium phosphate or calcium phosphate (13).

The exact mechanism of neobladder stone formation is not known as it has proved elusive to researchers to determine it. However, a variety of underlying etiologies may contribute to this condition, such as metabolic, infectious, structural, or idiopathic factors (14, 15). Urease-producing microorganisms (such as *Proteus mirabilis*, *Pseudomonas aeruginosa*, and *Klebsiella*) in urine infection can break down urea into ammonia and bicarbonate, which subsequently results in alkalinization of urine. Then, metabolic acidosis may be caused by neobladder-absorbed ammonia, all of which increase the risk of stones. Likewise, intermittent self-catheterization, mucus production, and urinary retention are independent risk factors for neobladder stone formation, leading to a stagnation of stone-forming factors and the entry of microorganisms into the urethra (16). Furthermore, foreign bodies (surgical titanium nails, non-absorbable sutures, etc.) can easily become the core of the stone, which is critical to the formation of stones (14, 17). However, one study showed that the stone formation rate in stapled orthotopic ileal neobladders was comparable to that reported in the literature for completely manually sutured ileal reservoirs (14).

Drug therapy is beneficial in preventing the formation of new stones by correcting underlying metabolic alterations and controlling infection. Doizi et al. (18) found that potassium citrate significantly increased urinary pH, potassium, and citrate levels and tended to lower urinary calcium; however, the long-term effects on the recurrence of calcium phosphate stones need further investigation. In another animal study, chlorthalidone was found to be more effective than potassium citrate in reducing calcium phosphate stones and improving bone quality in genetic hypercalciuric stone-forming rats (19). Drug therapy, especially antibiotic therapy, is an important treatment for infectious stones. The standardized application of antibiotics to persistent or recurrent urinary tract infections can effectively reduce the occurrence of stones in urinary diversions. Furthermore, considering that the neobladder will produce excessive mucus, keeping the bladder well empty is important to reduce the risk of stone recurrence and infection, but if it still occurs, it can be treated with adequate fluid intake (2 L or more), clean intermittent self-catheterization, and saline irrigation (20).

The main surgical approaches for bladder stones in patients with urinary diversion are represented by antegrade or retrograde ureteroscopic manipulation, open neocystolithotomy, or a combined approach. Ureteroscopic manipulation avoids the difficulties and complications of open surgical approaches, but the altered anatomy can make it a difficult procedure in this challenging patient population. For treating giant stones in the neobladder, as mentioned in our patient case and others, open neocystolithotomy for complete stone removal seems to be the only suitable and logical procedure due to the heavy stone burden involved.

Finally, follow-up and stone prevention in diverted patients with urolithiasis should be the primary goals, with particular attention to preventing urinary tract infections, correcting underlying metabolic abnormalities, and reducing siltation and mucus accumulation. The long-term complication incidence of radical cystectomy and neobladder formation cannot be ignored.

## Data Availability

The original contributions presented in the study are included in the article/Supplementary Material, further inquiries can be directed to the corresponding author.
